# The implications of increased mammographic breast density for breast screening in Jordan

**DOI:** 10.1002/jmrs.414

**Published:** 2020-06-23

**Authors:** Dana S. Al‐Mousa, Maram Alakhras, Kelly M. Spuur, Haytham Alewaidat, Mostafa Abdelrahman, Mohammad Rawashdeh, Patrick C. Brennan

**Affiliations:** ^1^ Faculty of Applied Medical Sciences Jordan University of Science and Technology Irbid Jordan; ^2^ School of Dentistry and Health Sciences Charles Sturt University Wagga Wagga NSW Australia; ^3^ Faculty of Health Sciences The University of Sydney Sydney NSW Australia

**Keywords:** Breast cancer, breast density, mammography, population health

## Abstract

**Introduction:**

Mammographic breast density is associated with a four to six times increased risk for breast cancer. Mammographic breast density varies by ethnicity, geographical region and age. The aim of this study was to document for the first time the mammographic breast density of Jordanian women and to explore its relationship with age.

**Methods:**

Mammograms completed at King Abdullah University Hospital (Irbid, Jordan) between January 2016 and August 2018 were retrospectively reviewed and classified for breast density using the American College of Radiology (ACR) Breast Imaging‐Reporting and Data System (BI‐RADS). Descriptive analyses and Kurskal–Wallis test were used to examine the association between age and mammographic breast density.

**Results:**

A total of 659 mammograms were reviewed. A significant inverse relationship was observed between age and breast density (*P* < 0.001). In women aged 40–49 years, 83.2% had dense breasts (ACR BI‐RADS (c) and (d)). This percentage decreased to 59.8% of women aged 50–59 years; 38.4% of women in their 60s and 37.9% of women aged 70 years or older (ACR BI‐RADS (c) only).

**Conclusion:**

The mammographic breast density of Jordanian women has been shown to be high across all age groups. Increased mammographic breast density is associated with increased breast cancer risk and renders mammography a less effective technique for the early detection of breast cancer. Breast cancer screening of Jordanian women should be individualised to develop screening protocols and include additional adjunct imaging to best manage women at high risk.

## Graphical abstract

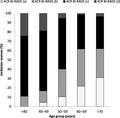

## Introduction

Breast cancer accounts for almost 1 in 4 cancer cases amongst women worldwide.^1^ Breast cancer is the leading cause of cancer deaths and the most commonly diagnosed cancer amongst women, with nearly 2.1 million newly diagnosed cases in 2018.^1^ In Jordan, a Middle Eastern Asian country, breast cancer is the most frequently diagnosed cancer and constituted 36.4% of all reported female cancers in 2018.^2^ The distribution of breast cancer by age group showed that most of the cases (30%) were diagnosed in the age group 40–49 years and 24.9% in the age group 50–59.^3^ According to Jordan National Cancer Registry report in 2012, 70% of breast cancer cases are diagnosed at advanced stages (III‐IV), during which prognosis is low and mortality rates are high.^4^ Women diagnosed with breast cancer at stage III and IV have reported mortality rates of 60.4% and 94.2% within five years of treatment, respectively.^5^

Mammography is the gold standard screening modality for the early detection of breast cancer, and it has been shown to significantly reduce breast cancer mortality.^6^ A study by Sankatsing et al.^6^ observed a mortality reduction of 30% amongst women aged 55–74 and 34% in women aged 75–79 attributed to breast screening. However, high breast density is known to decrease the ability of radiologists to detect breast carcinoma because the dense areas on the mammogram can mask or obscure subtle breast lesions, decreasing the efficacy of mammography as a diagnostic tool.^7^, ^8^ It has been shown that mammographic sensitivity decreases from 89.2% in low to 67.9% in high mammographic density.^7^ Mammographic specificity significantly decreases with increasing mammographic density ranging from 97.5% in women with low mammographic density and 91.5% in those with high mammographic density.^7^ Whilst ultrasound has always been the key adjunct modality for imaging of the dense breast, magnetic resonance imaging (MRI) and newer technology such as digital breast to mosynthesis (DBT) may also be utilised. Adjunct ultrasound imaging has demonstrated increased sensitivity (77.5%) compared with mammography alone (50%).^9^ However, adding ultrasound to mammography, in terms of positive predictive values (PPVs) has been shown to decrease from 23% in mammography alone to 11.9% when combined.^10^ By adding MRI to mammography, PPVs increased from 23% in mammography alone to 42.1% when combined.^10^ In addition, including DBT as an adjunct tool with mammography has been reported to result in significantly higher PPVs (24.1%) compared with mammography alone (13.0%).^11^

Mammographic breast density is a strong independent risk factor for breast cancer and women with high density breasts have four to six times increased risk of developing breast cancer as compared to women with low density (fatty) breasts.^12^ Different classification systems have been developed to both categorise breast density and describe the association between breast density and increased risk of breast cancer. The first system developed was Wolfe’s parenchymal patterns: N pattern (fatty breast), P1 (linear densities in less than one‐fourth) and P2 (linear densities in more than one‐fourth), and DY (extensive fibroglandular tissue “dysplasia’).^13^ Tabar’s classification system followed and described five types of mammographic parenchymal patterns (MPP): Grade I (scalloped contours with some lucent areas of fatty tissue), II (almost entirely of lucent areas of fatty tissue), III (fatty breast with retroareolar residual fibrous tissue), IV (predominantly nodular and linear densities throughout the breast) and V (homogeneous extensive fibrosis).^14^ Later, the American College of Radiology Breast Imaging Reporting and Data System (ACR BI‐RADS) was established for standardisation of mammographic reporting. The ACR BI‐RADS Atlas 2013 (5th version) is the updated version of the 2003 Atlas.^15^ It describes density as follows: (a) the breasts are almost entirely fatty; (b) there are scattered areas of fibroglandular density; (c) the breasts are heterogeneously dense, which may obscure small masses and (d) the breasts are extremely dense, which lowers the sensitivity of mammography.^15^ The inclusion of information concerning breast density in mammographic reporting improves the physician’s understanding of individual patient breast cancer risk and the sensitivity of mammography for that patient.

Breast density can be influenced by several other risk factors for breast cancer. It has been reported that breast density increased by 3–5% after oestrogen and progestin hormone treatment.^16^ Typically reflecting reproductive life, mammographic breast density varies between different age groups, generally decreasing with increased age. Younger premenopausal women evidence greater breast density than those who have undergone the menopausal changes of breast involution. Mammographic breast density and age are therefore important predictors of the accuracy of mammography. A study by Heidinger et al.^17^ reported that sensitivity of digital mammography increased with age from 72.1% in women aged 50–54 years to 82.8% in women aged 65–69 years.

Mammographic breast density underlies many international differences in breast cancer prevalence and is known to vary amongst different ethnic groups and geographical regions.^18^, ^19^. Knowledge related to mammographic breast density in a certain population is therefore crucial for planning an efficient screening programme especially where increased breast density is a known condition of the population. The trend of breast density reporting has mainly been derived from Western countries^19^ and other regions including Asia^20^ and South America.^21^

There is a paucity of information describing the mammographic density profile of the women in Jordan. Current imaging approaches in Jordan are based on what is known from the data of Western countries, highlighting the efficiency of their screening programmes. In designing an effective screening programme in Jordan, the mammographic breast density amongst Jordanian women and its relationship with age needs to be established. With breast cancer incidence the highest amongst Jordanian women aged 40–49 years, the target age groups of breast screening services of most developed countries starting at 50 years of age are unlikely to be appropriate.

This study is the first to evaluate the mammographic density profile of Jordanian women and the relationship between age and breast density in Jordan.

## Methods

Institutional review board approval was granted from the Jordan University of Science and Technology (Project Number 20170023). A retrospective review of all mammograms performed between January 2016 and August 2018 using a Mammorex Peruru MGU‐1000A digital mammography unit (Toshiba Medical Systems Corporation, Tokyo, Japan), at the King Abdullah II University Hospital (Irbid, Jordan)was undertaken. Each case was anonymised. All cases with a standard two‐view mammogram series (cranio‐caudal and medio‐lateral oblique projections) of both breasts were included. If additional projections were performed, only cranio‐caudal and medio‐lateral oblique projections were used to assess mammographic breast density. Cases with incomplete examination of both breasts, breast implants and mastectomy were excluded.

Mammographic breast density was defined according to the ACR BI‐RADS 5th version.^15^ Density was determined by three expert radiologists who are subspecialised in mammography, each with more than 10 years experience. All three radiologists use ACR BI‐RADS for categorising mammographic density in their daily clinical practice. Discordant classification was determined by the consensus rating (two of three readers).

Radiologists classified mammographic breast density in a 18‐m^2^ room, and walls were painted with light grey and brown matte paint to maintain minimal specular reflection. The ambient lighting was set to 20 lux at the position of the reader using a calibrated photometer (Model Konica Minolta CL‐200, Ramsey, NJ, USA). Mammograms were displayed on 5‐megapixel RADI FORCE GX540 (EIZO, Ishikawa, Japan) monitors, which were calibrated in accordance to Digital Imaging and Communications in Medicine (DICOM) standard.

To measure inter‐reader agreement between radiologists for classifying mammographic breast density, Cohen’s kappa statistics and 95% confidence intervals (CIs) were used. Kappa value was explicated as the following: 0.0–0.2 as slight agreement, 0.21–0.41 as fair agreement, 0.41–0.60 as moderate agreement, 0.61–0.80 as substantial agreement and 0.81–0.99 as almost perfect agreement.

To examine the association between age and breast density, descriptive analyses were performed. Kurskal–Wallis and Spearman’s non‐parametric statistical tests were conducted to evaluate the correlation between age and breast density. All analyses were performed using SPSS software (version 20.0, SPSS), with *P* values < 0.05 deemed to be statistically significant.

## Results

### Mammographic breast density classification inter‐reader agreement

The inter‐reader agreement between the three radiologists was almost perfect agreement with kappa value of 0.93 (95% CI: 0.90–0.96). All comparisons between paired radiologists showed almost perfect agreement; radiologists 1 and 2 (kappa value: 0.94, 95% CI: 0.89–0.98), radiologists 1 and 3 (kappa value 0.91, 95% CI: 0.86–0.96) and radiologists 2 and 3 (kappa value 0.93, 95% CI: 0.88–0.95).

### Age and mammographic breast density

A total of 659 mammograms were reviewed with a median age of 49 years (25th percentile, 44 and 75th percentile, 55 years). The majority of women, 375 (56.9%) were classified as ACR BI‐RADS (c) (heterogeneously dense). The distribution of women in each mammographic density category is described in Table 1.

**Table 1:** Mammographic breast density distribution according to ACR BI‐RADS grading and age

Mammographic breast density	*N*	Median age in years	25th age percentile in years	75th age percentile in years	Minimum age in years	Maximum age in years
ACR BI‐RADS (a)	58	57.00	51.75	64.75	42	86
ACR BI‐RADS (b)	144	53.00	49.00	58.00	53	77
ACR BI‐RADS (c)	375	48.00	43.00	53.00	32	85
ACR BI‐RADS (d)	82	44.00	40.75	48.00	32	72

The study population was categorised into five age groups, the highest frequency was (37.8%) in age group between 40 and 49 years. The results of the distribution of breast density patterns with age group in the study population are summarised in Table 2.

**Table 2:** Distribution of mammographic breast density with age group (*n* = 659)

Age group	ACR BI‐RADS				Total
	(a)	(b)	(c)	(d)	
<40	‐	9 (11.0%)	53 (64.6%)	20 (24.4%)	82 (12.4%)
40–49	11 (4.4%)	31 (12.4%)	160 (64.3%)	47 (18.9%)	249 (37.8%)
50–59	25 (10.5%)	71 (29.7%)	130 (54.4%)	13 (5.4%)	239 (36.3%)
60–69	13 (21.7%)	24 (40.0%)	22 (36.7%)	1 (1.7%)	60 (9.1%)
>70	9 (31.0%)	9 (31.0%)	10 (34.5%)	1 (3.4%)	29 (4.4%)
Total	58 (8.8%)	144 (21.9%)	375 (56.9%)	82 (12.4%)	659 (100%)

**Figure 1:**
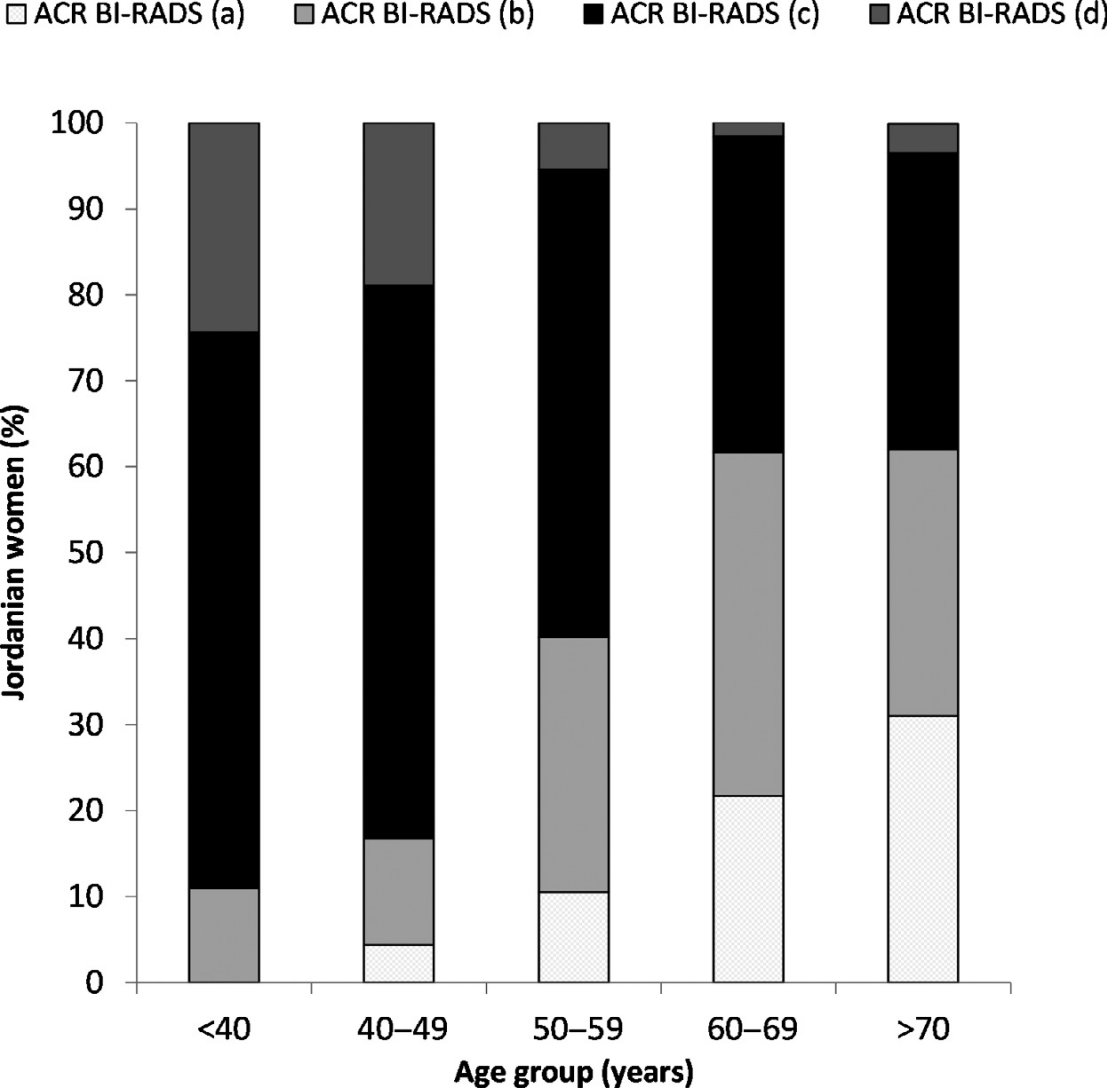
Age and mammographic breast density as defined by ACR BI‐RADS.

Using Kurskal–Wallis test, there was a significant inverse relationship demonstrated between age and breast density overall (*r* = −0.397, *P* < 0.001) as shown in Fig. 1.

## Discussion

This is the first study documenting the mammographic density of Jordanian women and evaluating the relationship between density and age. Since breast density is known to vary between women of different age groups, ethnicity and geographical region, it is crucial to design screening programmes that reflect the needs specific to the population they support. Current screening guidelines in Jordan have been adapted from the National Cancer Comprehensive Network, United States of America (USA). The guidelines do not take into account the breast density profile of Jordanian women and its relationship to age which until now has remained undocumented.

It is well established that mammographic breast density is reflective of reproductive life and typically decreases with increasing age.^22^ The current study documents as expected, a significant inverse relationship between age and breast density amongst Jordanian women with mammographic breast density decreasing as Jordanian women age. However, the current study also reveals that the majority of the Jordanian women reviewed across all ages were categorised as ACR BI‐RADS (c) (56.9%). This means that the majority of women who attend screening in Jordan have high‐density breasts and are at increased risk of breast cancer.

**Figure 2:**
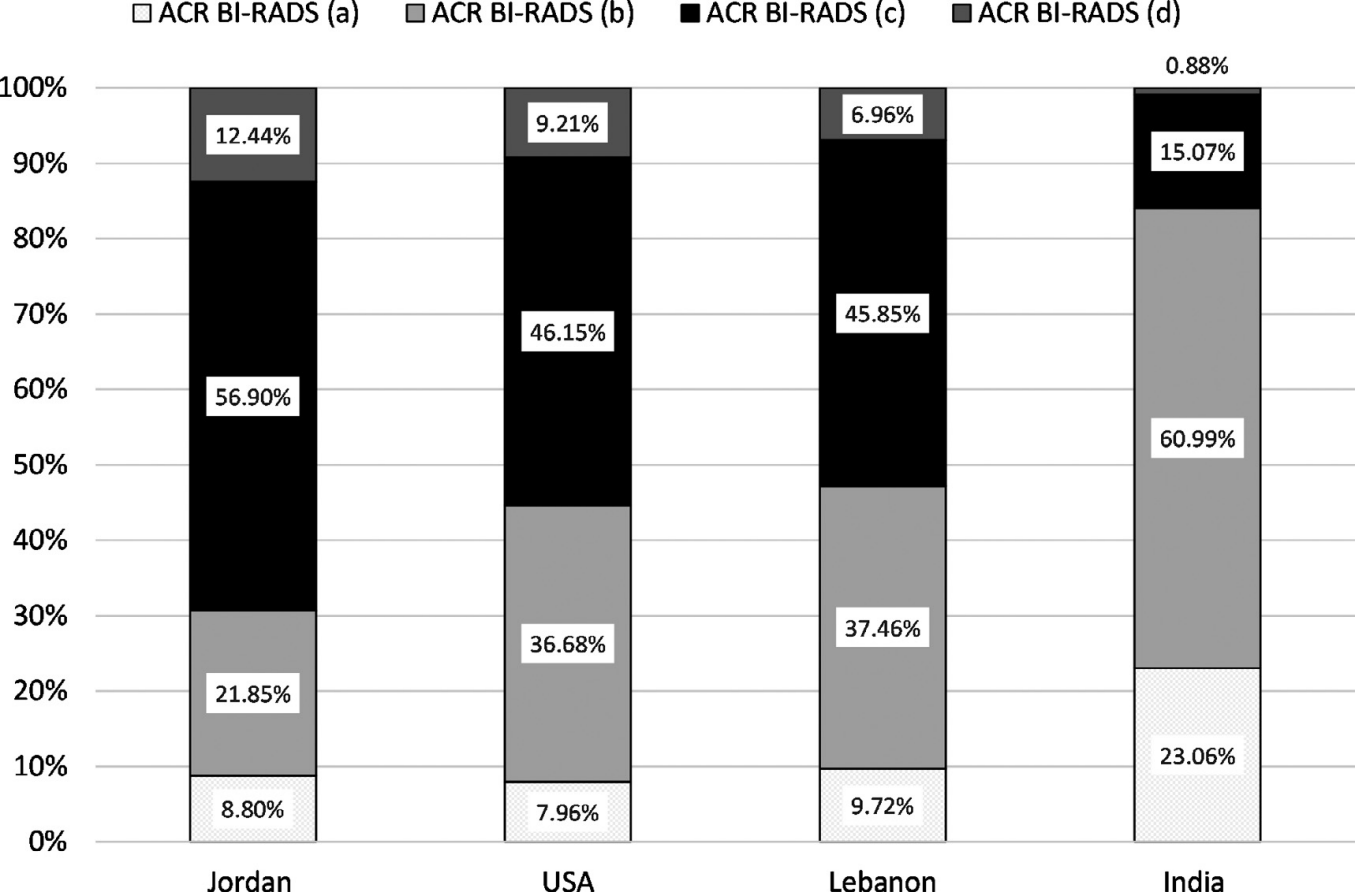
Mammographic breast density distribution of Jordanian, USA, Lebanese and Indian women.

The literature reports that mammographic sensitivity in 2D mammography decreases from 89.2% in low to 67.9% in high mammographic density^7^ due to the potential for density to mask subtle breast lesions (masking effect). Decreased sensitivity can lead to higher rates of interval cancers and decrease the efficacy of breast screening programmes.^7^, ^8^ The overall percentage of Jordanian women with high‐dense breasts (69.3%), that is with breasts categorised as ACR BI‐RADS (c) or (d), is greater than the USA (55.4%),^19^ Lebanon (52.9%)^23^ and India (16%)^18^ as shown in Fig. 2. This emphasises that the Jordanian screening programme must be developed according to local needs rather than simply through the adoption of international protocols.

The literature has shown that for women aged less than 50 years, rates of interval cancer increases from 19% for entirely fatty to 98% for the extremely dense breast.^8^ This study reports that 83.1 % of women aged 40–49 years had dense breasts (ACR BI‐RADS (c) and (d)); decreasing to 59.8% of women between 50–59 years and 38.4% of women in their 60s. Most of cancer cases amongst Jordanian women (30%) were in the age group 40–49 years and 24.9% in the age group 50–59.^3^ As increased breast density is associated with increased breast cancer risk and decreased mammographic sensitivity, it can be hypothesised that for the majority of Jordanian women undertaking screening, the use of mammography alone will not be an effective imaging modality. Individualised imaging protocols based on density and risk should be considered to maximise the potential for the early detection of breast cancer in this population.^19^ Although no clear guidelines have yet been developed, the incorporation of additional imaging modalities such as, ultrasound, MRI and DBT has shown to increase breast cancer screening programme sensitivity for women with elevated risk of breast cancer and high breast density.^9^, ^10^, ^24^

The routine addition of ultrasound to mammography in women with high mammographic density has been shown to increase sensitivity from 50% with mammography or ultrasound alone to 77.5% when combined.^9^ Therefore, for young women and women with high mammographic breast density, including ultrasound as an adjunct imaging tool will improve screening benefit by increasing cancer detection rates and decreasing interval cancer rates.^25^ Ultrasound is considered as a cost‐effective modality compared with other available modalities such as MRI.^26^ However, it should be noted that the use of ultrasound may also result in higher number of false positives.^9^ This will potentially lead to an increase in the number of biopsies, serious patient anxiety, women uncertainty andoverdiagnosis.^9^, ^26^

When combining MRI, with mammography for screening women of high risk of breast cancer, sensitivity increased to 93% compared with 33% of mammography alone.^10^ A study by Sardanelli et al.^27^ showed that for women with high‐density breasts, MRI has significantly higher sensitivity (81%) compared with mammography alone (60%). MRI as a screening tool may contribute to earlier detection of breast cancer particularly in high‐risk women and women with increased breast density.^28^ However, adopting MRI as a screening modality is currently limited to women at high risk of developing breast cancer because of its lower specificity (85.9%) compared with mammography (96.8%).^29^

Since the implementation of DBT, several studies have examined the combined effect of DBT and mammography.^24^, ^30^ For women with high mammographic breast density, the addition of DBT demonstrated significant improvement in radiologists’ performance (Area Under the receiver operating curve (AUC), 0.88) as compared to mammography alone (AUC, 0.79).^30^ In addition, including DBT as an adjunct tool with mammography has been reported to result in significantly higher PPVs, cancer detection rates and lower screening recall rates, in both women with low or high mammographic density^24^ because DBT has the ability to eliminate the masking effect.

Given the importance of the relationship between breast density and breast cancer risk, mammographic breast density notification laws should also be enacted. In the USA, 38 states have passed laws that mandate notification of mammographic breast density to women after mammographic imaging. Depending on patients’ individual risk factors, supplemental screening may be required.^31^

Another important consideration of this current work is regarding the appropriate age to start mammography screening and the most appropriate target age group. In Jordan, mammography screening guidelines currently allow screening to commence at the age of 40 years with women being allowed to return every year thereafter. The highest breast cancer incidence in Jordan is amongst women in the 40–49 years age group^3^ and in this study 83.1% of these women demonstrated dense breasts (ACR BI‐RADS (c) and (d)). Therefore, it is recommended to at least maintain the current starting age for screening and to examine the potential advantages of developing a screening protocol tailored to women with high mammographic breast density with additional imaging modalities. In addition, screening women every 12‐months interval seems appropriate to increase mammographic sensitivity amongst women of high risk and high mammographic breast density.^32^ However, a study by Miglioretti et al.^33^ projected that the annual screening of 100,000 women aged between 40 and 74 years would result in 125 radiation‐induced breast cancers and 16 deaths, compared with 968 breast cancer deaths prevented by early detection from screening. The increased density profile of Jordanian women combined with earlier and more frequent screening would increase the risk of radiation‐induced cancer and must be appropriately considered.

It should be noted that there are some limitations in this study. Women included in this study were only a subset of the population of the North and Middle areas in Jordan, a larger and more even representation of the population is needed in future studies. Another limitation is the sample distribution. The study reviewed 508 women aged 40–59 years but only 89 aged 60 years and over. If more equal numbers of women were used results may change. Additionally, the visual ACR BI‐RADS grading of mammographic breast density used in this study can be subjective. The potential for subjectivity should be reduced by the involvement of specialised and highly experienced radiologists. Although in this study radiologists demonstrated almost perfect agreement (kappa value of 0.93), it is possible that the high mammographic density rating of the Jordanian population is due to the threshold that the radiologists use which may have raised the mammographic breast density. Therefore, it should be acknowledged that it is more accurate to use automated methods.

## Conclusions

The findings of this study confirm the inverse relationship of a women’s age and mammographic breast density. The results suggest that a significant proportion of Jordanian women have high density ACR BI‐RADS (c) and (d) breasts, whereby they could potentially benefit from the use of additional imaging methods such as ultrasound, MRI or DBT. The current study emphasises the importance of developing individualised breast cancer screening guidelines, which are derived according to woman age, breast density, and overall risk for breast cancer. Further research is required to validate the results of this study.

## Conflict of Interest

The authors declare that they have no conflicts of interest.

## Data Availability

The data that support the findings of this study are available from the corresponding author upon reasonable request.
